# Relative effectiveness of azithromycin in killing intracellular Porphyromonas gingivalis

**DOI:** 10.1002/cre2.17

**Published:** 2016-02-05

**Authors:** Pin‐Chuang Lai, John D. Walters

**Affiliations:** ^1^ Division of Biosciences, College of Dentistry The Ohio State University Wexner Medical Center Columbus Ohio 43210 USA; ^2^ Division of Periodontology, College of Dentistry The Ohio State University Wexner Medical Center Columbus Ohio 43120 USA

**Keywords:** Antimicrobial agents, invasive microorganisms, periodontitis, pharmacokinetics

## Abstract

Invasive infections by *Porphyromonas gingivalis* are associated with persistent periodontal attachment loss and can be difficult to eliminate by scaling and root planing. Azithromycin (AZM) inhibits *P*. *gingivalis* and is actively accumulated by most human cells. We used an in vitro infection model to compare the effectiveness of AZM in killing intracellular *P*. *gingivalis* to the combined regimen of amoxicillin (AMX) and metronidazole (MET). Transport of [^3^H]‐AZM by human gingival fibroblasts was characterized. Monolayers of Smulow–Glickman gingival epithelial cells or gingival fibroblasts were infected with *P*. *gingivalis* (strain 33277 or W83). After extracellular bacteria were eliminated with teicoplanin, infected cells were treated with therapeutic concentrations of AZM, AMX, or AMX + MET. Viable intracellular bacteria were released by cell lysis and plated on blood agar for enumeration. Antimicrobial activity against planktonic *P*. *gingivalis* was also evaluated. While survival of intraepithelial *P*. *gingivalis* 33277 was not significantly different after treatment with the three regimens, survival in infected fibroblasts was significantly lower after AZM treatment (65.9 ± 5.5%) compared with AMX (92.2 ± 3.5%) or AMX + MET (79.8 ± 5.2%, *P* < 0.01). Carnitine, a competitive inhibitor of AZM transport, reduced killing by AZM by ~55% (*P* < 0.05). Survival of intrafibroblast *P*. *gingivalis* W83 was also significantly lower after AZM treatment compared with the other regimens (*P* < 0.05). At therapeutic concentrations, AZM was significantly more active against intracellular *P*. *gingivalis* than against planktonic *P*. *gingivalis* (*P* < 0.0083). Gingival epithelial cells and fibroblasts possess a transport system that accumulates AZM and enhances elimination of intracellular *P*. *gingivalis*. Compared with the combination of AMX and MET, AZM was equally effective against intraepithelial *P*. *gingivalis* 33277 and significantly more effective against both strains of *P*. *gingivalis* from infected gingival fibroblasts. The results suggest that AZM could be a reasonable alternative to the regimen of AMX and MET for periodontal patients who should not take these agents due to known side effects or compliance issues.

## Introduction

Periodontitis is an infectious disease resulting in inflammation within the supporting tissues of the teeth and progressive loss of attachment and bone (Flemmig, 1999). It reportedly affects 47.2% of US adults aged 30 years and older (Eke et al., 2012). In periodontitis, polymicrobial communities transition from physiologically compatible commensal bacteria to pathogenic entities with the presence of some keystone pathogens. The complex interactions among members of microbial communities and between the communities and host immune system can be explained through a polymicrobial synergy and dysbiosis model (Lamont and Hajishengallis, 2015). Control of subgingival bacterial biofilms through mechanical treatment (scaling and root planing) usually halts the progression of periodontal breakdown, but the responses are not favorable in some patients. Some keystone periodontal pathogens, including *Aggregatibacter actinomycetemcomitans* and *Porphyromonas gingivalis*, have the ability to invade pocket epithelium and underlining connective tissue, evading the host immune system and lingering in the periodontal tissue. This could partly explain the unresponsiveness and recurrence of periodontitis (Bragd et al., 1987). Although adjunctive systemic antibiotics are not routinely indicated in periodontal therapy, it is reasonable to use antibiotics that can cross cell membranes to control intracellular infections (Tulkens, 1990).

*Porphyromonas gingivalis* is a gram‐negative, non‐motile, obligate anaerobe, which exhibits a strong association with chronic periodontitis (Preus et al., 1995; Griffen et al., 1998). Its ability to invade gingival epithelium and fibroblasts makes it difficult to eliminate by conventional therapy (Sandros et al., 1994; Dogan et al., 2000). Its major virulence factors, including gingipains, fimbriae, lipopolysaccharide, and capsules, have also been attributed to dysregulation of the host immune‐inflammatory responses (Bostanci and Belibasakis, 2012). *P*. *gingivalis* has been used as a bacterial marker for progression of periodontitis (van Winkelhoff et al., 2002).

Azithromycin (AZM) is a macrolide antibiotic derived from erythromycin. Its properties include broad‐spectrum activity against most species of periodontal pathogens, a plasma half‐life of over 65 h, ability to be actively taken up by mammalian cells, and more sustained concentrations in gingival tissues and gingival crevicular fluid than in serum (Zuckerman, 2000; Jain and Danziger, 2004; Lai et al., 2011). Its antimicrobial activity against *P*. *gingivalis* has been confirmed through in vitro and clinical studies (Pajukanta, 1993; Goldstein et al., 1999; Haffajee et al., 2008; Oteo et al., 2010). Adjunctive use of systemic AZM with scaling and root planing results in significantly more reduction of probing depth and gain of clinical attachment in patients with chronic or aggressive periodontitis (Mascarenhas et al., 2005; Haas et al., 2008; Oteo et al., 2010).

The combination of amoxicillin (AMX) and metronidazole (MET) is the most commonly used antibiotic regimen in periodontal therapy and is effective in the treatment of both chronic and aggressive periodontitis (Keestra et al., 2015a; Keestra et al., 2015b). Neither AMX nor MET is concentrated inside human cells (Mandell and Coleman, 2001; Yu et al., 2009). Our laboratory has previously shown that intracellular AZM accumulation helps eliminate *A*. *actinomycetemcomitans* from infected gingival epithelial monolayers (Lai and Walters, 2013). Because no previous study has directly compared AZM with the combined regimen, we used a similar approach to test the hypothesis that AZM is as effective at killing intracellular *P*. *gingivalis* in gingival fibroblasts as AMX plus MET. The association between intracellular AZM accumulation and intracellular activity was also examined.

## Materials and Methods

### Cell culture

Smulow–Glickman (SG) gingival epithelial cells derived from human attached gingiva (Smulow and Glickman, 1966) were maintained in Dulbecco's modified Eagle's medium (Invitrogen, Grand Island, NY) supplemented with 10% heat‐inactivated fetal bovine serum (FBS; Atlanta Biologicals, Norcross, GA). A human gingival fibroblast strain harvested as previously described (Mariotti and Cochran, 1990) was cultured in minimal essential medium (Invitrogen) containing 2 mM l‐glutamine and 10% heat‐inactivated FBS. Both cell lines were fed every 3 days and cultured to confluent monolayers at 37°C in the presence of 5% CO_2_ until a confluent monolayer was formed.

### Bacterial culture

*Porphyromonas gingivalis* 33277 (American Type Culture Collection, Manassas, VA) and a well‐characterized laboratory strain (W83) were grown in Trypticase soy broth (Becton, Dickinson and Company, Sparks, MD) supplemented with 1 mg/mL yeast extract (Gibco, Grand Island, NY), 5 µg/mL hemin (Sigma‐Aldrich, St. Louis, MO) and 1 µg/mL menadione (Sigma‐Aldrich) at 37°C in an anaerobic chamber (Lamont et al., 1995).

### Antibiotics

All targeted antibiotics, including AZM, AMX, MET, and moxifloxacin (MFX), were from Sigma‐Aldrich. Teicoplanin was acquired from Selleck Chemicals LLC (Houston, TX). The MIC of each antibiotic against planktonic *P*. *gingivalis* was determined by broth dilution methods (Stalons and Thornsberry, 1975). Briefly, overnight cultures of *P*. *gingivalis* were resuspended in TSB supplemented with yeast extract, hemin, and menadione to an optical density of 0.001 at 600 nm, which corresponds to 10^6^ CFU/mL. *P*. *gingivalis* was then cultured anaerobically for 48 h to determine the lowest concentration, which prevents visible growth of bacteria (MIC).

### Assays of azithromycin transport

Confluent gingival fibroblast monolayers were washed with Hank's balanced salt solution (HBSS; Invitrogen), harvested by brief treatment with 0.25% trypsin ethylenediaminetetraacetic acid (Invitrogen) and suspended in HBSS at a density of 10^6^ cells/mL. AZM transport was assayed by measuring changes in cell‐associated radioactivity over time (Pascual et al., 1997). Aliquots of cell suspension were incubated at 37°C with [^3^H]‐AZM (American Radiolabeled Chemicals, St. Louis, MO) at a concentration of 10 µg/mL for time course assays and at 10–50 µg/mL for kinetic assays to determine the Michaelis constant (*Km*) and maximal velocity of transport (Vmax). After the indicated interval (1–30 min for uptake time course assays and 3 min for kinetic assays), 0.5‐mL aliquots of cell suspension were rapidly withdrawn, layered over 0.3 mL of a mixture of canola oil‐dibutyl phthalate (3:10), and centrifuged for 30 sec at 15,000 × *g* in a microcentrifuge (Walters et al., 1999). After removal of the aqueous and oil layers, the cell pellets were recovered, lysed by agitation in 1 mL of sterile water, and subjected to liquid scintillation counting. The intracellular volume was measured by incubation with [^3^H]‐water (5 μCi/mL; NEN Life Science Products, Boston, MA) for 20 min at 37°C. Volume determinations were corrected for extracellular water trapped in the pellet, which was determined by incubation under the same conditions with [^14^C]‐inulin (1 μCi/mL; PerkinElmer, Waltham, MA) (Brayton et al., 2002).

To observe the efflux of AZM, gingival fibroblasts were loaded to a steady‐state intracellular AZM concentration by incubation for 20 min at 37°C in HBSS containing 10 g/mL [^3^H]‐AZM. To trigger efflux of intracellular AZM stores, we abruptly diluted AZM in the extracellular medium 11:1 with 37°C HBSS. The decrease in intracellular AZM concentration was monitored for 60 min.

### In vitro infection model

The protocol of infection assays was modified from Lamont et al. (1995). In brief, confluent SG epithelial cell and gingival fibroblast monolayers in 24‐well culture plates were washed twice with HBSS. *P*. *gingivalis* from overnight culture were harvested, resuspended in cell culture medium specific to the corresponding host cell lines, and laid over monolayers to infect at a 100 bacteria to one host cell ratio for 90 min. Afterward, extracellular bacteria were removed by HBSS washes and treatment with 100 µg/mL teicoplanin for 1 h. The monolayers were again washed four times with HBSS, and an aliquot of the final wash was plated on reduced blood agar (Anaerobic systems, Morgan Hill, CA) to ensure that HBSS washes and treatment with teicoplanin removed viable extracellular bacteria (Eick et al., 2004). Infected cell monolayers were then cultured in the presence of AMX (4 µg/mL), AMX (4 µg/mL) + MET (10 µg/mL), and AZM (8 µg/mL), which correspond to therapeutic concentrations attainable in gingival crevicular fluid, for 4 h (van Winkelhoff et al., 1996). As controls, infected monolayers were also cultured under identical condition in the absence of any antibiotic, and in the presence of 3.2 µg/mL MFX (Eick et al., 2004). At the end of incubation, monolayers were washed four times with HBSS and lysed in sterile water to release intracellular *P*. *gingivalis*. Dilutions of the lysate were plated on reduced blood agar plates for the enumeration of surviving colony‐forming units. Data were expressed as a percentage of the colonies recovered from the controls without antibiotics.

To determine whether the intracellular accumulation of AZM enhances the killing of intracellular *P*. *gingivalis*, antibiotic efficiency against planktonic bacteria was tested under similar conditions and compared with that against intracellular bacteria. In addition, an AZM transport inhibitor (L‐carnitine) was added to the epithelial model to evaluate its influence on killing of *P*. *gingivalis* (Lai and Walters, 2013).

## Results

### Azithromycin transport by gingival fibroblasts

**Figure 1:**
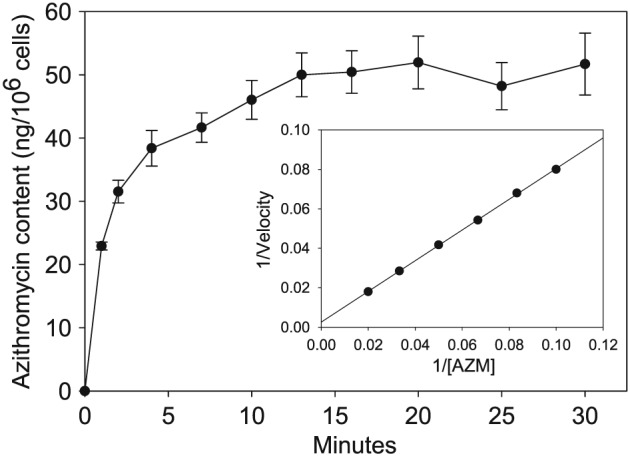
Time course of azithromycin (AZM) uptake in gingival fibroblasts. Transport was initiated by the addition of [^3^H]‐AZM to aliquots of suspended cells. Changes in cell‐associated radioactivity over time were subsequently determined by liquid scintillation counting. All data are expressed as the mean ± standard error of the mean of six experiments. The Inset is as representative Linewearver–Burk plot of the initial phase of AZM transport (0–3 min) by the gingival fibroblasts.

**Figure 2:**
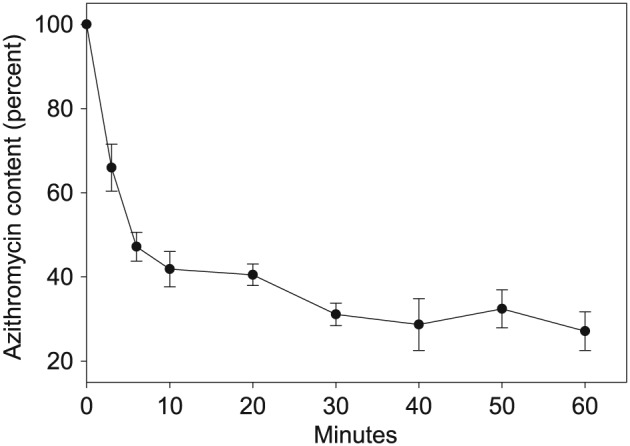
Efflux of [^3^H]‐AZM from loaded gingival fibroblasts. Gingival fibroblasts were loaded to steady state by incubation in medium containing 10 µg/mL [^3^H]‐AZM for 20 min. Efflux was triggered by diluting extracellular antibiotic medium 11‐fold with 37°C HBSS. All data are expressed as the mean ± standard error of the mean of three experiments. For references, 100% content corresponds to 60 ng/10^6^ cells.

Gingival fibroblasts rapidly took up AZM, reaching the point of saturation after 15 min (Fig. 1). When gingival fibroblasts were incubated in medium containing clinically attainable concentrations of AZM for 20 min, the steady‐state intracellular concentrations were at least 10‐fold higher than the extracellular concentrations (Table 1). The observed AZM transport activity exhibited Michaelis–Menten kinetics, and kinetic analysis indicated the gingival fibroblasts internalized AZM with an estimated K_m_ of 262 ± 12.0 µg/mL and a maximum velocity (V_max_) of 394 ± 39.0 ng/min/10^6^ cells (Fig. 1 inset). The efflux assays indicated that AZM transport is bi‐directional. Once the AZM concentration in the medium was diluted 11‐fold; gingival fibroblasts lost approximately 33% of their AZM content within 3 min and almost 60% after 20 min. Thereafter, the rate of efflux decreased dramatically. At the end of the 60‐min observation, the gingival fibroblasts retained approximately 30% of their original steady‐state AZM content (Fig. 2).

**Table 1:** Azithromycin cellular/extracellular concentration ratios in Smulow–Glickman epithelial cells and gingival fibroblasts.

Extracellular concentration (µg/mL)	Gingival epithelium^a^	Gingival fibroblast
0.5	N/A	10.64 ± 1.99
2	21.74 ± 1.56	10.90 ± 1.46
5	23.66 ± 0.97	11.19 ± 1.80
10	18.34 ± 2.44	10.67 ± 1.85

All data are expressed as the mean ± standard error of the mean of three experiments.

a Data adapted from a previous publication with permission (Lai and Walters, 2013).

### Killing of Porphyromonas gingivalis 33277 by selected antibiotics

**Figure 3:**
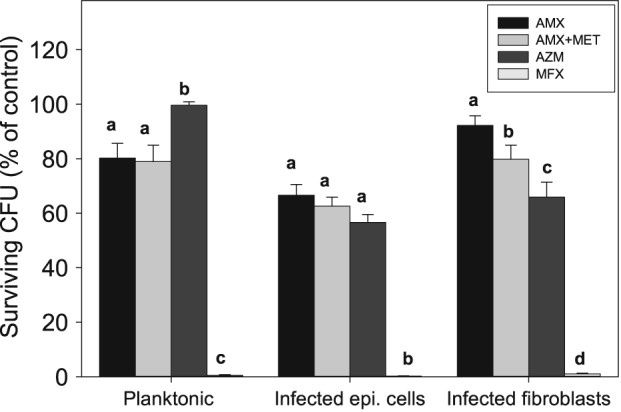
Killing of planktonic, intraepithelial, and intrafibroblast *Porphyromonas gingivalis* 33277 by AZM. Smulow–Glickman epithelial cell or gingival fibroblast monolayers infected by *P*. *gingivalis* 33277, or planktonic bacteria suspended in medium were treated with 8 µg/mL azithromycin, 4 µg/mL amoxicillin, 4 µg/mL amoxicillin + 10 µg/mL metronidazole, or 3.2 µg/mL moxifloxacin. Monolayers incubated in antibiotic‐free medium were used as negative control. Intracellular *P*. *gingivalis* were released by cell lysis, plated on reduced blood agar, and enumerated. Data were presented as percentage of the colonies recovered from the negative controls. The data represent the mean ± standard error of the mean of at least four experiments. Within each cluster, significantly different effects among regimens are denoted by different letters (*P* < 0.05, Holm–Sidak test).

After 4‐h incubation under conditions to similar to the infection assays, AMX and AMX + MET produced comparable killing on planktonic *P*. *gingivalis* 33277 (~19% and ~22% of the control, respectively). AZM showed little effect, while the positive control, MFX, killed more than 99% of planktonic bacteria. The differences between AZM and the other regimens were statistically significant (*P* < 0.01, Holm–Sidak test) (Fig. 3).

All tested antibiotic regimens showed different levels of killing against intrafibroblast *P*. *gingivalis* 33277 (Fig. 3). AZM killed approximately 34% of the *P*. *gingivalis* in infected gingival fibroblasts, while AMX and AMX + MET killed ~8% and ~20%, respectively. Approximately 99% of the intrafibroblast bacteria were killed by MFX over the same period. The differences among all regimens were statistically significant (*P* < 0.05, Holm–Sidak test). It appeared that AZM was significantly more effective against intrafibroblast *P. gingivalis* 33277 than against planktonic bacteria (~34% vs ~0.5%, *P* < 0.0083, *t* test, after adjustment), but this phenomenon was not observed from either AMX or AMX + MET.

As for intraepithelial *P*. *gingivalis* 33277, all tested antibiotic regimens showed significant suppression of intracellular bacteria (*P* < 0.05, Holm–Sidak test). Killing of intraepithelial *P*. *gingivalis* was ~43%, ~38%, and ~33% of the control colony‐forming units after treatment with AZM, AMX + MET, and AMX, respectively (Fig. 3). Meanwhile, MFX killed more than 99% of intraepithelial *P*. *gingivalis*. The differences among AMX, AMX + MET, and AZM failed to reach statistical significance (*P* > 0.05). Only AZM exhibited significantly greater killing of intraepithelial *P*. *gingivalis* relative to planktonic *P*. *gingivalis* (*P* < 0.0083, *t*‐test, adjusted).

A separate series of transport assays in which 2 mM L‐carnitine and 4 µg/mL AZM were co‐incubated with SG epithelial cells for 4 h indicated carnitine reduced the steady‐state intracellular AZM concentration from 92.0 µg/mL to ~68.5 µg/mL (data not shown). Carnitine alone reduced approximately 10% of intraepithelial *P*. *gingivalis* 33277, whereas AZM alone killed ~30% of the bacteria. In the presence of carnitine, more than half of the killing effect produced by AZM was reversed (*P* < 0.05, Holm–Sidak test, data not shown). The difference in effects produced by carnitine alone and in combination with AZM was not statistically significant (*P* > 0.05).

### Killing of Porphyromonas gingivalis W83

**Figure 4:**
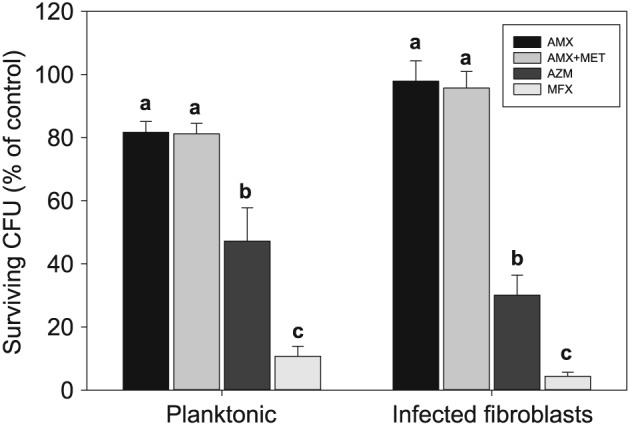
Killing of planktonic and intrafibroblast *Porphyromonas gingivalis* W83 by azithromycin (AZM). Data were presented as percentage of the colonies recovered from the controls. The data represent the mean ± standard error of the mean of at least seven experiments. Within each cluster, significantly different effects among regimens are denoted by different letters (*P* < 0.05, Holm–Sidak test).

The levels of inhibition on planktonic *P*. *gingivalis* W83 generated from AMX and AMX + MET were comparable (~18% and ~19%, respectively). In contrast to its activity against the 33277 strain, AZM killed ~53% of planktonic *P*. *gingivalis* W83, which is significantly more active than the other two regimens (*P* < 0.01, Holm–Sidak test). The positive control (MFX) killed approximately 90% of bacteria (Fig. 4).

A similar trend was observed with killing of intrafibroblast *P*. *gingivalis* W83. AZM eliminated approximately 70% of the control *P*. *gingivalis* W83, whereas AMX and AMX + MET had no significant effect on intracellular bacteria (~2% and ~4%, respectively, Fig. 4). The antimicrobial activity of AZM against intrafibroblast W83 was significantly greater than that of AMX or AMX + MET (*P* < 0.01, Holm–Sidak test). In addition, AZM produced significantly stronger antimicrobial activity against intrafibroblast W83 than against their planktonic counterparts (*P* < 0.0167, *t*‐test, adjusted).

## Discussion

Our data indicate that human gingival fibroblasts take up and accumulate relatively high intracellular concentrations of AZM through an active transport system. When gingival fibroblasts were cultured in medium containing 5 µg/mL AZM, the steady‐state intracellular AZM concentration was approximately 55.8 µg/mL. Intracellular stores of AZM appear to move out of fibroblasts when the extracellular concentration drops, which could help maintain high concentrations in tissue fluid. SG gingival epithelial cells used in this study also have an active transport system that exhibits similar characteristics and concentrates AZM more than 20 times inside (Lai and Walters, 2013). Human neutrophils also have an active transport system for AZM (Gladue et al., 1989; Pascual et al., 1995). To date, only one pharmacokinetic study has been conducted in human fibroblasts (Gladue and Snider, 1990); however, some of the observed characteristics in the current study were different from those found in human skin fibroblasts. AZM uptake by human skin fibroblasts was reportedly continuous over a 3‐day period, leading to extracellular/cellular concentration ratios of 174 and 3,738 after 1 and 72 h, respectively. The differences could be partly explained by different cell origins and incubation conditions (adherent versus suspended cells). It has been reported that AZM concentration in lysosomes changes lysosomal pH and function in phagocytes (Nujic et al., 2012). The maximal non‐cytocidal concentration of AZM for a human gingival epithelial cell line NDUSD‐1 was reported to be 10 μM (Inoue et al., 2004). It is reasonable to expect that uptake reaches a point of saturation once intracellular AZM starts to affect cellular functions. Given their large numbers in gingival connective tissue, the ability to take up and retain AZM could allow gingival fibroblasts to function as “reservoirs” that maintain high tissue concentrations (Malizia et al., 1997; Jain et al., 2012).

The antimicrobial activities of the targeted antibiotics were compared under similar aerobic conditions. Although all antibiotics were active against *P*. *gingivalis* 33277, as judged by the MIC values tested in our laboratory (<0.03, <0.015, and 0.5 µg/mL for AMX, MET, and AZM, respectively), their activity against planktonic bacteria was rather modest. This could be partially explained by the experimental conditions. When exposed to normal aerobic tissue culture atmosphere, *P*. *gingivalis*, an obligate anaerobe, typically synthesizes proteins and enzymes such as heat shock proteins and superoxide dismutase for survival (instead of proteins for multiplication) (Meuric et al., 2008). In addition, the medium used for these assays did not contain hemin and menadione and was less supportive of bacterial growth (Marcotte and Lavoie, 1998). AMX, which inhibits peptidoglycans cross‐linking (Drawz and Bonomo, 2010), may not work efficiently when bacteria are not actively replicating. MET is a prodrug that has no antimicrobial effects before its reduction by anaerobic enzyme systems such as pyruvate : ferredoxin oxidoreductase and NADPH nitroreductases (Edwards, 1993; Trend et al., 2001). Under our experimental conditions, there may not have been sufficient reduced intermediate to interact effectively with bacterial DNA. AZM inhibits bacterial growth by binding to the 50S ribosomal subunit of the bacterial ribosome (Champney and Miller, 2002). It is interesting that we did not observe any antimicrobial activity against planktonic *P*. *gingivalis*, considering its potential to arrest synthesis of enzymes and proteins that help *P*. *gingivalis* survive. In contrast, MFX (3.2 µg/mL) almost completely eliminated planktonic *P*. *gingivalis* within 4 h. MFX is a fluoroquinolone antibiotic and exerts its bactericidal effect mainly through inhibition of DNA gyrase, which is required for DNA replication, transcription, and repair (Blondeau and Hansen, 2001). The effective and efficient killing by MFX is consistent with a previous study (Ardila et al., 2010).

Our results suggest that AZM is more effective at killing intrafibroblast *P*. *gingivalis* 33277 than AMX or AMX + MET. Compared with its weak activity against planktonic *P*. *gingivalis*, AZM appears to benefit substantially from its cellular accumulation by gingival fibroblasts. AZM exhibits concentration‐dependent killing kinetics, in which efficacy can be predicted by the area under the concentration–time curve over 24 h (AUC) divided by the MIC (Gunderson et al., 2001). The active transport system of fibroblasts concentrates AZM, leading to an elevated AUC/MIC ratio and greater bacterial inhibition. In contrast, the intracellular concentrations of AMX and MET have been shown to be equal to or less than the extracellular concentrations (Mandell and Coleman, 2001; Yu et al., 2009). This, along with environmental limitations mentioned in the previous section, could explain why AMX and AMX + MET did not exhibit improved activity against intrafibroblast *P*. *gingivalis* 33277. In general, the results observed with *P*. *gingivalis* W83 were similar to those seen with strain 33277, except for the greater suppression produced by AZM. Comparison of the antimicrobial activities against planktonic and intrafibroblast W83 indicated that cellular accumulation enhanced killing of bacteria.

Our study indicated that active transport by SG gingival epithelial cells enhanced AZM's antimicrobial activity against intracellular *P*. *gingivalis* 33277, and this enhancement could be reversed by an inhibitor of the transporter. Based on our previous study, some organic cations including quinidine and L‐carnitine competitively inhibit AZM transport by oral and gingival epithelial cells (Lai and Walters, 2013). The relationship between intracellular AZM concentrations and antimicrobial activity is also evident in other studies. Addition of L‐carnitine reduced killing of intracellular *A*. *actinomycetemcomitans* to 54% of that produced by AZM alone in SG gingival epithelial cells (Lai and Walters, 2013). In a macrophage model, verapamil (an inhibitor of P‐glycoprotein efflux pump) increased intracellular AZM concentrations by ~2.4‐fold and enhanced the activity against *Listeria monocytogenes* and *Staphylococcus aureus* when low extracellular concentrations were used (Seral et al., 2003). Human neutrophils loaded with AZM exhibited an antimicrobial effect significantly greater than the sum of suppression produced by AZM or neutrophils alone. This benefit was most pronounced when bacteria vastly outnumbered neutrophils (Lai et al., 2015).

It is interesting to observe differences in the relative effectiveness of AZM compared with the other antibiotics. AZM is more effective against intrafibroblast *P*. *gingivalis* but is only equally effective in the epithelial cell model. We speculate that gingival epithelial cells could provide a more hospitable environment for *P*. *gingivalis* growth than gingival fibroblasts, thereby enhancing the bactericidal effects of AMX and AMX + MET. There could be several factors that, alone or together, make epithelial cells a better environment in which to survive and replicate. It is evident that epithelial cells provide some levels of protection compared with the extracellular environment (Houalet‐Jeanne et al., 2001; Hosogi and Duncan, 2005). The host anti‐oxidative mechanisms could potentially create a permissive environment (Madianos et al., 1996). In addition, *P*. *gingivalis* may respond to epithelial cells and fibroblasts differently after their initial contact and invasion into these cells. Genes involved with the oxidative stress response and heat shock proteins are up‐regulated significantly during infection of HEp‐2 human epithelial cells (Hosogi and Duncan, 2005). Although similar studies have not previously been conducted in fibroblasts, significant differences in the behavior of *P*. *gingivalis* could potentially make them more susceptible to antibiotics in epithelial cells.

The limitations of this study are mainly related to differences between our experimental model and in vivo conditions. Because periodontitis is a response to polymicrobial biofilm, our focus on a single pathogen in a planktonic state is an oversimplified approach. The aerobic culture conditions used in the model were adjusted to optimize the viability of human cells, but these conditions limit the growth of *P*. *gingivalis*. For this reason, the in‐vitro model could only be used for relatively brief periods of antibiotic treatment.

The role cell invasion by bacteria plays in the pathogenesis of periodontitis is controversial. While bacterial invasion and spreading from cell to cell has been proposed as a possible mechanism in periodontal and cardiovascular diseases (Deshpande et al., 1998; Dorn et al., 1999; Saito et al., 2012), the effectiveness of AMX + MET in periodontal clinical trials suggests that it is not necessary to target intracellular bacteria to obtain an adjunctive benefit. The results of this study do not detract from the clinical effectiveness of the AMX + MET combination. Although the observed antimicrobial activity of AZM against *P*. *gingivalis* in our study was modest (killing only 20% to 40% of control CFU), it is comparable with the activity of MET, clindamycin, and doxycycline reported in a previous in vitro study (Eick et al., 2004). To put these results into context, the favorable clinical and microbiological outcomes reported in clinical studies were the result of antibiotic regimens of at least 1 week in duration, while the present in‐vitro study was limited to only 4 h of antibiotic treatment.

## Conclusions

Our data suggest that human gingival fibroblasts possess an active transport system that accumulates AZM at intracellular concentrations at least 10‐fold higher than those in the extracellular environment. This accumulation enhances the antimicrobial activity of AZM against intracellular *P*. *gingivalis* and makes it more effective than the combination of AMX + MET in eliminating the bacteria from infected gingival fibroblasts. In gingival epithelial cells, AZM was equally effective in eliminating *P*. *gingivalis* 33277. It is reasonable to prescribe AZM instead of AMX + MET in periodontal applications for patients who are allergic to β‐lactam drugs or are hypersensitive to the side effects of AMX or MET. In theory, it should be easier to obtain a high degree of compliance with AZM compared with the combined regimen. Within the limitation of this study, the data suggest that AZM is a reasonable alternative to AMX + MET when prescribing a systemic antibiotic to treat *P*. *gingivalis*‐associated periodontitis.

## Conflict of Interest

The authors have no conflicts of interest to declare.
